# A Trivariate Extreme Value Distribution Applied to Flood Frequency Analysis

**DOI:** 10.6028/jres.099.035

**Published:** 1994

**Authors:** Carlos A. Escalante-Sandoval, Jose A. Raynal-Villasenor

**Affiliations:** Engineering Graduate Studies Division, Universidad Nacional Autonoma de Mexico, 04510 Mexico, DF, Mexico; Institute of Sciences, Universidad Autonoma de Chihuahua, 31800 Chihuahua, Chih., Mexico

**Keywords:** distribution functions, distribution models, flood frequency analysis, method of maximum likelihood, trivariate extreme value distributions

## Abstract

A trivariate extreme value distribution has been derived from the logistic model for the multivariate extreme value distribution. The construction of its corresponding probability distribution and density function is described. In order to obtain the parameters of such a trivariate distribution, a generalized maximum likelihood estimation procedure is described to allow for the cases of samples with different record lengths. Furthermore the reliability of the estimated parameters of the irivariate extreme value distribution is measured through the use of relative information ratios. A region in Northern Mexico with six gauging stations has been selected to apply the trivariate model. Results produced by the proposed model have been compared with those obtained by general extreme value (GEV) distribution functions.

## 1. Introduction

Flood frequency analysis has been carried out by using univariate distribution functions, the extreme value distributions being an important set of distributions used in this field of study. Generally, parameters of such distributions are estimated from a short record of flows. The variability of these estimates has prompted exploration of joint estimation models which use information from streamflow records of neighboring gauging stations.

In pioneering papers Finkelstein [1], Tiago de Oliveira [2], and Gumbel [3] gave the foundations for the multivariate approach to extreme value distributions. Following this work, several bivariate extreme value models began to appear in the literature. Rueda [4] explored the logistic and mixed models for bivariate extreme value distributions when both marginals are extreme value type I (EVI) distributions. He reported improvements in the estimation of parameters when the bivariate approach is used. Raynal [5] developed and applied three bivariate options from the logistic model of bivariate extreme value distribution for flood frequency analysis. He found that there exists an improvement in the parameter estimation phase, even in the case when both samples have the same record lengths.

Herein, the trivariate approach of multivariate extreme value distribution is presented with a view to its application to flood frequency analysis.

General characteristics, the procedure for estimation, and reliability of parameters of the trivariate extreme value distributions will be described in the following sections. An actual application of the proposed model to six gauging stations in Northern Mexico is presented in the paper.

## 2. Characteristics of the Trivariate Logistic Model

From the multivariate extension of the logistic model for bivariate extreme value distribution [3], the trivariate approach is:

$$\begin{array}{c} F(x,y,z,\underline{\theta })=\exp\{-[{\left(\ln\;F(x)\right)}^{m}\\ +{\left(-\ln\;F(y)\right)}^{m}+{\left(-\ln\;F(z)\right)}^{m}{\}}^{1/m}, \end{array}$$ (1)

where *m* is the association parameter (*m* ≥ 1) and *F*(*s*) = *F*(*s*, *θ*) is the marginal distribution function of *s*. Equation (1) must satisfy the following inequalities (Tiago de Oliveira [6, 7]):

$$\begin{array}{c} F(x)F(y)F(z)\leq F(x,y,z)\leq \min[F(x),F(y),F(z)]\\ {\left[F(x,y,)F(x,z)F(y,z)\right]}^{\frac{1}{4}} \end{array}$$ (2)

$$\leq F(x,y,z)\leq \frac{{\left[F(x,y)F(x,z)F(y,z)\right]}^{1/2}}{{\left[F(x)F(y)F(z)\right]}^{1}}.$$ (3)

Marginals in Eq. (1) can be either EVI distributions:

$$F(s)=\exp\left(-\exp-\left(\frac{s-u}{\alpha }\right)\right)$$ (4)

or GEV distributions:

$$F(s)=\exp-{\left(1-\left(\frac{s-u}{\alpha }\right)\beta \right)}^{1/\beta }.$$ (5)

The combinations have been named (Escalante [8]):

- Trivariate extreme value distribution type 111 (TEV111) or TriGumbel distribution. All marginals are EVI distributions.
- Trivariate extreme value distribution type 112 (TEV112) or BiGumbel-GEV distribution.
- Trivariate extreme value distribution type 122 (TEV122) or BiGEV-EVI distribution.
- Trivariate extreme value distribution type 222 (TEV222) or TriGumbel distribution. All marginals are GEV distributions.

The particular form of Eq. (1), when the marginals are GEV distributions for the maxima, is (Escalante [8]):

$$\begin{array}{c} F(x,y,z,u_{1},\alpha_{1},\beta_{1},u_{2},\alpha_{2},\beta_{2},u_{3},\alpha_{3},\beta_{3},m_{t})=\\ \exp[-(\left(1-\left(\frac{x-u_{1}}{\alpha_{1}}\right)\beta_{1}\right){\;}^{m_{2}/\beta_{1}}\\ +\left(1-\left(\frac{y-u_{2}}{\alpha_{2}}\right)\beta_{2}\right){\;}^{m_{1}/\beta_{2}}\\ {+\left(1-\left(\frac{z-u_{3}}{\alpha_{3}}\right)\beta_{3}\right){\;}^{m_{t}/\beta_{3}})}^{1/m_{1}}], \end{array}$$ (6)

where *μ_i_*, *α_i_* and *β_i_*, *i* = 1, 2, 3, are the location, scale and shape parameters of the marginal GEV distributions for the maxima. The corresponding probability density function is (Escalante [8]):

$$\begin{array}{c} f(x,y,z,u_{1},\alpha_{1},\beta_{1},u_{2},\alpha_{2},\beta_{2},u_{3},\alpha_{3},\beta_{3},m_{1})=\frac{1}{\alpha_{1}\alpha_{2}\alpha_{3}}{\left(1-\left(\frac{x-u_{1}}{\alpha_{1}}\right)\beta_{1}\right)}^{m_{1}/\beta_{1}-1}{\left(1-\left(\frac{y-u_{2}}{\alpha_{2}}\right)\beta_{2}\right)}^{m_{1}/\beta_{2}-1}\\ {\left(1-\left(\frac{z-u_{3}}{\alpha_{3}}\right)\beta_{3}\right)}^{m_{1}/\beta_{3}-1}\exp\left[-{\left({\left(1-\left(\frac{x-u_{1}}{\alpha_{1}}\right)\beta_{1}\right)}^{m_{1}/\beta_{1}}+{\left(1-\left(\frac{y-u_{2}}{\alpha_{2}}\right)\beta_{2}\right)}^{m_{1}/\beta_{2}}+{\left(1-\left(\frac{z-u_{3}}{\alpha_{3}}\right)\beta_{3}\right)}^{m_{1}/\beta_{3}}\right)}^{1/m_{1}}\right]\\ {\left[{\left(1-\left(\frac{x-u_{1}}{\alpha_{1}}\right)\beta_{1}\right)}^{m_{1}/\beta_{1}}+{\left(1-\left(\frac{y-u_{2}}{\alpha_{2}}\right)\beta_{2}\right)}^{m_{1}/\beta_{2}}+{\left(1-\left(\frac{z-u_{3}}{\alpha_{3}}\right)\beta_{3}\right)}^{m_{1}/\beta_{3}}\right]}^{1/m_{1}-3}[\left(1-m_{1}\right)\left(1-2_{m_{1}}\right)\\ +{\left({\left(1-\left(\frac{x-u_{1}}{\alpha_{1}}\right)\beta_{1}\right)}^{m_{1}/\beta_{1}}+{\left(1-\left(\frac{y-u_{2}}{\alpha_{2}}\right)\beta_{2}\right)}^{m_{1}/\beta_{2}}+{\left(1-\left(\frac{z-u_{3}}{\alpha_{3}}\right)\beta_{3}\right)}^{m_{1}/\beta_{3}}\right)}^{2/m_{1}}+\left(3m_{1}-3\right)\\ {\left({\left(1-\left(\frac{x-u_{1}}{\alpha_{1}}\right)\beta_{1}\right)}^{m_{1}/\beta_{1}}+{\left(1-\left(\frac{y-u_{2}}{\alpha_{2}}\right)\beta_{2}\right)}^{m_{1}/\beta_{2}}+{\left(1-\left(\frac{z-u_{3}}{\alpha_{3}}\right)\beta_{3}\right)}^{m_{1}/\beta_{3}}\right)}^{1/m_{1}}]. \end{array}$$ (7)

## 3. Estimation of Parameters

The method of maximum likelihood for estimating the parameters of trivariate extreme value distributions has been chosen due to its characteristics for consistency in large sample estimation and applicability in estimating the parameters of cumbersome density functions.

For the ease of trivariate distribution functions, the sample arrangements could allow having either an equal or different record length in any of the samples to be analysed.

In order to consider all possible combinations of data, it is required to have a sufficiently flexible formulation, therefore the following general form of the likelihood function will be used based on the generalization obtained by Anderson [9]:

$$\begin{array}{c} L\left(x,y,z,\underline{\theta }\right)={\left[\overset{n_{1}}{\underset{i=1}{\Pi }}f\left(p_{i},{\underline{\theta }}_{1}\right)\right]}^{l_{1}}{\left[\overset{n_{2}}{\underset{i=1}{\Pi }}f\left(p_{i},q_{i},{\underline{\theta }}_{2}\right)\right]}^{l_{2}}\\ {\left[\overset{n_{3}}{\underset{i=1}{\Pi }}f\left(x,y,z,{\underline{\theta }}_{3}\right)\right]}^{I_{3}}{\left[\overset{n_{4}}{\underset{i=1}{\Pi }}f\left(r_{i},s_{i},{\underline{\theta }}_{4}\right)\right]}^{l_{4}}{\left[\overset{n_{5}}{\underset{i=1}{\Pi }}f\left(r_{i},{\underline{\theta }}_{5}\right)\right]}^{l_{5}}, \end{array}$$ (8)

where:

*n*_1_, *n*_2_: = are respectively the univariate and bivariate record lengths before the common period *n*_3_,
*n*_4_, *n*_5_: = are respectively the bivariate and univariate record lengths after the common period *n_3_*,
*p*: = is the variable with univariate record before the common period,
(*p, q*): = are the variables with bivariate record before the common period,
(*x, y, z*): = are the variables with trivariate record during the common period,
(*r, s*): = are the variables with bivariate record after the common period,
*r*: = is the variable with univariate record after the common period,
*I_i_*: = are indicator numbers such that: *I_i_* = 1 if *n_i_* > 0 and *I_i_* = 0 if *n_i_* = 0.

The logarithmic function will be used instead of the likelihood function. So, Eq. (8) is transformed into:

$$\begin{array}{c} LL\left(x,y,z,\underline{\theta }\right)=I_{1}\left[\sum_{l=1}^{n_{1}}\ln\;f\left(p_{i},{\underline{\theta }}_{1}\right)\right]\\ +I_{2}\left[\sum_{l=1}^{n_{2}}\ln\;f\left(p_{i},q_{i},{\underline{\theta }}_{2}\right)\right]+I_{3}\left[\sum_{i=1}^{n_{3}}\ln\;f\left(x,y,z,{\underline{\theta }}_{3}\right)\right]\\ +I_{4}\left[\sum_{l=1}^{n4}\ln\;f\left(r_{i},s_{i},{\underline{\theta }}_{4}\right)\right]+I_{5}\left[\sum_{i=1}^{n_{5}}\ln\;f\left(r_{i},{\underline{\theta }}_{5}\right)\right]. \end{array}$$ (9)

The maximum likelihood estimators of parameters for the trivariate extreme value distributions are those values for which Eq. (9) is maximized.

The corresponding logarithmic likelihood function for the trigeneral extreme value (TEV222) distribution function, based on Eq. (9) from [8] is shown in Eq. (10):

LL(x,y,z,u1,α1,β1,u2,α2,β2,u3,α3,β3,mt,mb1,mb1)=I1{−n1lnαp+∑i=1n1[−(1−(pi−upαp)βp)1/βp+ln(1−(pi−upαp)βp)1/βp−1]}+I2{∑i=1n2[−(lnαp+lnαp)+ln(1−(pi−upαp)βp)mb1/βp−1+ln(1−(qi−uqαq)βq)mb1/βq−1+ln((1−(pi−upαp)βp)mb1/βp+(1−(qi−uqαq)βq)mb1/β0)1/mb1−2+ln((mb1−1)+((1−(pi−upαp)βp)mb1/βp+(1−(qi−uqαq)βq)mb1/βq)1/mb1)−((1−(pi−upαp)βp)mb1/βp+(1−(qi−uqαq)βq)mb1/βq)1/mb1]}+I3{−n3(lnα1+lnα2+lnα3)+∑i=1n3[ln(1−(xi−u1α1)β1)mt/β1−1+ln(1−(yi−u2α2)β2)mt/β2−1+ln(1−(zi−u3α3)β3)mt/β3−1+ln((1−(xi−u1α1)β1)mt/β1+(1−(yi−u2α2)β2)mt/β2+(1−(zi−u3α3)β3)mt/β3)1/mt−3+ln[(1−mt)(1−2mt)+((1−(xi−u1α1)β1)mt/β1+(1−(yi−u2α2)β2)mt/β2+(1−(zi−u3α3)β3)mt/β3)2/mt+(3mt−3)((1−(xi−u1α1)β1)mt/β1+(1−(yi−u2α2)β2)mt/β2+(1−(zi−u3α3)β3)mt/β3)1/mt]−((1−(xi−uiα1)β1)mt/β1+(1−(yi−u2α2)β2)mt/β2+(1−(zi−u3α3)β3)mt/β3)I/mt]}+I4{∑i=1n4[−(lnα1+lnαq)+ln(1−(ri−urαr)βr)mb2/β1−1+ln(1−(si−usαs)βs)mb2/β2−1+ln((1−(ri−urαr)βr)mb2/βr+(1−(si−usαs)βs)mb2/βs)1/mb2−2+ln((mb2−1))((1−(ri−utαr)βt)mb2/βr+(1−(si−usαs)βs)mb2/βs)1/mb2)−((1−(ri−urαr)βr)mb2/βi+(1−(si−usαs)βs)mb2/β3)1/mb2/β3]}+I5{−n5lnα1+∑i=1n5[−(1−(ri−urαr)βr)1/βr+ln(1−(ri−uiαr)βt)1/βr−1]} (10)

where;

*m_t_*: trivariate association parameter
*m*_b1_, *m*_b2_: bivariate association parameter before and after the common period, respectively.

$\sum_{i=1}^{n_{1}}\ln\;f\left(p_{i},{\underline{\theta }}_{1}\right)$ and $\sum_{i=1}^{n_{5}}\ln\;f\left(r_{i},{\underline{\theta }}_{5}\right)$ take the form:

$$\sum_{i=1}^{n_{j}}\left[-\ln\;\alpha_{t}-{\left(1-\left(\frac{t_{i}-u_{t}}{\alpha_{1}}\right)\beta_{t}\right)}^{1/\beta_{t}}+\ln{\left(1-\left(\frac{t_{i}-u_{1}}{\alpha_{1}}\right)\beta_{t}\right)}^{1/\beta_{t}-1}\right].$$ (11)

Similarly, $\sum_{i=1}^{n_{2}}\ln\;f\left(p_{i},q_{i},{\underline{\theta }}_{2}\right)$ and $\sum_{i=1}^{n_{4}}\ln\;f\left(r_{i},s_{i},{\underline{\theta }}_{4}\right)$ take the following form (bivariate relationship with both GEV marginals):

$$\begin{array}{c} \sum_{i=1}^{n_{1}}[-\left(\ln\;\alpha_{t}+\ln\;\alpha_{w}\right)+\ln\left(1-\left(\frac{t_{i}-u_{t}}{\alpha_{t}}\right)\beta_{t}\right){\;}^{m_{2}/\beta_{1}-1}\\ +\ln\left(1-\left(\frac{w_{i}-u_{w}}{\alpha_{w}}\right)\beta_{w}\right){\;}^{m_{b}/\beta_{w}-1}\\ +\ln(\theta \left(1-\left(\frac{t_{l}-u_{1}}{\alpha_{t}}\right)\beta_{t}\right){\;}^{m_{b}/\beta_{1}}\\ {+\left(1-\left(\frac{w_{i}-u_{w}}{\alpha_{w}}\right)\beta_{w}\right){\;}^{m_{b}/\beta_{w}})}^{1/m_{b}-2}\\ +\ln(\left(m_{b}-1\right)+(\left(1-\left(\frac{t_{l}-u_{t}}{\alpha_{t}}\right)\beta_{1}\right){\;}^{m_{b}/\beta_{t}}\\ {+\left(1-\left(\frac{w_{i}-u_{w}}{\alpha_{w}}\right)\beta_{w}\right){\;}^{m_{b}/\beta_{n}})}^{1/m_{b}})\\ -(\left(1-\left(\frac{t_{i}-u_{1}}{\alpha_{t}}\right)\beta_{t}\right){\;}^{m_{b}/\beta_{t}}\\ {+\left(1-\left(\frac{w_{i}-u_{w}}{\alpha_{w}}\right)\beta_{w}\right){\;}^{m_{b}/\beta_{w}})}^{1/m_{b}}]. \end{array}$$ (12)

Given the complexity of the mathematical expressions in Eq. (10) and their partial derivatives with respect to the parameters, the constrained multivariable Rosenbrock method, Kuester and Mize [10], was applied to obtain the maximum likelihood estimators for the parameters by the direct maximization of Eq. (10). The required initial values of the parameters to start the optimization of Eq. (10) were provided by the univariate maximum likelihood estimators of the parameters for the case of the location, scale, and shape parameters. The initial values of the association parameters, bivariate and trivariate, were set equal to 2, following the procedure developed by Escalante [8].

## 4. Reliability of Estimated Parameters

The indicator selected to measure the reliability of estimated parameters when using the trivariate distribution as compared with the univariate counterpart was the asymptotic relative information ratio.

Table 1 shows a sample of relative information ratios obtained by using the following set of parameters:

$$\begin{array}{lll} u_{1}=15.0, & \alpha_{1}=2.0, & \beta_{1}=-0.20\\ u_{2}=12.0, & \alpha_{2}=1.2, & \beta_{2}=-0.15\\ u_{3}=10.0, & \alpha_{3}=1.0, & \beta_{3}=-0.10 \end{array}$$

## 5. Case Study

A region located in Northern Mexico, with a total of six gauging stations, was selected to apply the proposed methodology to the flood frequency analysis. Tables 2–4 show the results of the application of the trivariate extreme value distributions for the maxima to the data recorded in such gauging stations.

In order to compare the goodness of fit between the univariate and trivariate maximum likelihood estimates of the parameters in stations considered in the case study, the standard error of fit, as defined by Kite [11], was obtained and is displayed in Table 5.

## 6. Conclusions

The logistic model for trivariate general extreme value distribution for the maxima has been proposed. Asymptotic and data base results suggest that the proposed model is a suitable option to be considered when performing flood frequency analysis.

## Figures and Tables

**Table 1:** Asymptotic relative information ratios of the parameters of the TEV222 distribution for the maxima *n*_3_ = 25; *m*_t_ = 2; *m*_b2_ = 2

Parameter	*n*_4_	0	25	50	75
*μ*_2_	0	1.0868	1.3695	1.5055	1.5856
	25	1.4460	1.5942	1.6823	
	50	1.6295	1.7201		
	75	1.7408			
*α*_1_	0	1.0141	1.2274	1.3256	1.3821
	25	1.2712	1.3753	1.4356	
	50	1.3941	1.4555		
	75	1.4662			
*β*_1_	0	1.2405	1.3555	1.4041	1.4312
	25	1.3864	1.4382	1.4671	
	50	1.4514	1.4806		
	75	1.4883			
*μ*_2_	0	1.0876	1.3722	1.5094	1.5903
	25	1.0599	1.2263	1.3333	
	50	1.0500	1.1683		
	75	1.0450			
*α*_2_	0	1.0135	1.2151	1.3065	1.3587
	25	0.9835	1.1046	1.1788	
	50	0.9734	1.0604		
	75	0.9684			
*β*_2_	0	1.2442	1.3469	13901	1.4140
	25	1.1324	1.2069	1.2507	
	50	1.0934	1.1507		
	75	1.0736			
*μ*_3_	0	1,0882	1.0462	1.0302	1.0217
	25	1.0604	1.0417	1.0313	
	50	1.0506	1.0390		
	75	1.0736			
*α*_3_	0	1.0126	0.9553	0.9334	0.9218
	25	0.9822	0.9549	0.9395	
	50	0.9790	0.9542		
	75	0.9669			
*β*_3_	0	1.2437	1.0268	0.9535	0.9166
	25	1.1314	1.0246	0.9704	
	50	1.0923	1.0223		
	75	1.0724			

**Table 2:** Correlation coefficients and relative sample sizes for the triplets of stations for the case study

Tripleis of stations	Correlation coefficient	Relative sample sizes				
		*n*_1_	*n*_2_	*n*_3_	*n*_4_	*n*_5_
Acatitan-Sta Cruz-Ixpalino	0.926	9	2	26	0	0
Choix-Huitcs-Sn Francisco	0.969	0	14	18	7	0

**Table 3:** Univariate maximum likelihood estimates of the parameters of the GEV distributions defined by the data of the gauging stations of the case study

Station	Location	Scale	Shape
Acatitan	576.21	283.80	−0.62
Choix	236.69	130.15	−0.12
Huites	1564.78	978.87	−0.57
Ixpalino	772.57	473.97	−0.38
Sn Francisco	926.53	532.56	−0.65
Sta Cruz	835.74	440.23	−0.40

**Table 4:** Trivariate maximum likelihood estimates of the parameters of the TEV222 distribution defined by the data of the gauging stations of the case study

Station	Location	Scale	Shape
Acatitan	568.93	269.44	−0.64
Choix	220.85	128.2V	−0.39
Huites	1603.30	1038.53	−0.68
Ixpalino	795.03	490.86	−0.46
Sn Francisco	943.69	540.73	−0.67
Sta Cruz	850.97	467.74	−0.52

**Table 5:** Standard errors of fit for gauging stations of case study

Station	Standard error of fit	
	Univariate (GEV)	Trivariate (TEV222)
Acatitan	244.40^a^	253.90
Choix	87.70	58.80^a^
Huites	1024.00	831.90^a^
Ixpalino	537.90	393.00^a^
Sn Francisco	350.80^a^	401.50
Sta Cruz	497.20	259.60^a^

a Minimum standard error of fit.
